# Novel Photo-Driven Activated Enzyme–Titanium Nanobiohybrids for Photocatalytic Applications

**DOI:** 10.3390/nano16130823

**Published:** 2026-07-04

**Authors:** Francesca Palla, Carla Garcia-Sanz, Marzia Marciello, Jose M. Palomo

**Affiliations:** 1 Instituto de Catálisis y Petroleoquímica (ICP), CSIC, c/Marie Curie 2, 28049 Madrid, Spain; francesca.palla2@unibo.it (F.P.); c.garcia.sanz@csic.es (C.G.-S.); 2 Nanobiotechnology for Life Sciences Laboratory, Department of Chemistry in Pharmaceutical Sciences, Faculty of Pharmacy, Universidad Complutense de Madrid (UCM), Plaza Ramón y Cajal s/n, 28040 Madrid, Spain; marmarci@ucm.es; 3 Microscopy and Dynamic Imaging Unit, Fundación Centro Nacional de Investigaciones Cardiovasculares Carlos III (CNIC), Calle Melchor Fernández Almagro 3, 28029 Madrid, Spain

**Keywords:** titanium, nanobiohybrids, photocatalysis, remediation, brookite

## Abstract

This work reports the development of innovative enzyme–titanium nanobiohybrids synthesized via a protein-assisted approach to obtain efficient and sustainable photocatalysts for environmental remediation. By addressing the limitations of conventional TiO_2_ nanoparticle synthesis, this strategy enables controlled material properties under milder, potentially scalable conditions for enhanced ROS-driven degradation of persistent dye pollutants. This work employs a bio-assisted synthesis approach using β-glucosidase as a protein scaffold, TiCl_4_ as the titanium precursor, and H_2_O_2_ in bicarbonate buffer at room temperature, eliminating the need for harsh conditions and high temperatures. The biological moiety guides the nanoparticle formation, controlling size and morphology while preventing aggregation, all performed under mild conditions. X-ray diffraction determined that the Ti hybrid was composed of TiO_2_ brookite species. TEM analyses demonstrated the formation of well-dispersed nanostructures of around 700 nm. The resulting nanobiohybrids showed excellent photocatalytic activity, achieving >99% Rhodamine B degradation under UV light in only 1 h compared to visible light. The catalyst was capable of degrading Rhodamine B at a concentration approximately 36 times above the recommended threshold for water. Furthermore, a preactivation of the catalyst by direct exposition of it to UV-395 nm light greatly enhanced the efficiency in the photocatalytic process, being inactive in visible light. The Ti–enzyme hybrid showed excellent recyclability over five consecutive cycles and retained good activity after storage, demonstrating its stability. This study introduces a sustainable and efficient route for synthesizing Ti-based nanobiohybrids, providing a promising strategy for advanced photocatalytic applications in water treatment and environmental remediation.

## 1. Introduction

Water pollution has long represented one of the most relevant environmental challenges. Pollutants and contamination may originate from natural processes, but the predominant sources are anthropogenic, including the discharge of untreated industrial effluents and improper disposal of household wastes [1]. Both inorganic and organic contaminants are typically present in polluted water. Many of these compounds are persistent in the environment, bioaccumulative, and resistant to conventional treatment processes, holding a significant risk to human and ecosystem health [2,3,4,5,6,7]. Among the various strategies developed, particular attention has been given to processes based on the generation of highly reactive oxygen species (ROS), such as hydroxyl radicals (•OH) and superoxide anions (O_2_•^−^), which are able to degrade a wide spectrum of organic contaminants [8]. Within this context, photocatalytic degradation employs semiconductors, such as TiO_2_, CdS, and ZnO, that, upon irradiation with adequate wavelengths, generate ROS capable of efficiently oxidizing and breaking pollutants in aqueous environments [9]. Among the available photocatalysts, titanium dioxide (TiO_2_) stands out for its peculiar physicochemical properties, including high redox potential, excellent chemical and thermal stability, non-toxicity, low cost, natural abundance and a wide band gap with efficient charge carrier mobility. Chemically, titanium dioxide (TiO_2_) exists in three main forms, anatase, rutile, and brookite, which have distinct structural, electronic, and catalytic properties. Upon exposure to near-UV light, electrons in the valence band are excited to the conduction band, leaving behind positively charged holes (h^+^). Among the three, anatase is widely recognized as the most photoactive phase. This superior performance is attributed to its favorable charge carrier dynamics, higher surface area and enhanced interfacial chemistry. Although rutile is the thermodynamically most stable across a broad range of temperatures and pressures, its photocatalytic activity is generally lower than that of anatase [10,11,12,13]. Comparatively, brookite has been much less studied because of the difficulty of its synthesis in pure form. However, in recent years, the interest in brookite has increased. The number of papers on preparation and photocatalytic activity of brookite is increasing. Brookite is, however, now recognized as an active phase, with potential enhanced performance with respect to anatase [14,15,16,17]. However, the large-scale application of TiO_2_-based photocatalysts still faces significant limitations. Although TiO_2_ has been extensively investigated, a problem resides in the fact that key properties of TiO_2_ nanoparticles, such as morphology, surface area, crystal structure, degree of crystallinity, and band gap, are strongly dependent on the synthesis method employed [13]. Among these, thermal treatment represents a crucial parameter in determining TiO_2_ characteristics, including crystallinity, porosity, and morphology. In particular, calcination at temperatures above 400 °C is typically necessary to remove organic residues and promotes the transformation of amorphous TiO_2_ into crystalline anatase. Carefully controlled heating rates and calcination times seem to be essential to preserve surface properties while achieving the desired crystal structure [14]. Therefore, a wide range of synthesis strategies for TiO_2_ nanoparticles have been reported; however, many of these approaches present issues in terms of scalability, cost, and energy used. As a result, developing synthetic methodologies that are simple, efficient, cost-effective, and sustainable remains a critical challenge for advancing TiO_2_-based photocatalysis towards applications.

A particularly promising route to overcome these limitations, which is the central strategy of this work, is the design of bio-nanohybrid systems that exploit biomolecules, such as proteins or enzymes, as both structural and functional scaffolds. This bio-assisted approach exploits the three-dimensional architecture and rich surface chemistry of proteins, whose functional groups, including amine, carboxyl, hydroxyl, and thiol moieties, act as natural coordination and nucleation centers for metal ions, enabling the controlled in situ formation of nanostructures under mild aqueous conditions at room temperature [18]. Their three-dimensional structures create specific metal coordination environments that facilitate biomineralization processes and enable the formation of monodisperse, stable nanoparticles or other nanostructures [18,19,20]. This allows precise control over nanoparticle size and morphology while simultaneously preventing aggregation. Compared to conventional synthesis methods, this approach offers several clear advantages: milder reaction conditions, shorter reaction times, enhanced structural control, and an inherently greener and more sustainable process that eliminates the need for harsh processing conditions [18,19,20]. Several groups have demonstrated that protein and peptide scaffolds can template the synthesis of TiO_2_ nanoparticles with controlled morphology, crystal phase, and photocatalytic activity [21,22,23,24]. However, these approaches still rely on calcination steps at high temperatures to achieve the desired crystalline phase, retaining one of the key limitations of conventional TiO_2_ synthesis. A more general and versatile strategy was introduced by us, developing the concept of nanobiohybrids, in which isolated commercial enzymes are used as scaffolds to directly induce the in situ formation of metal nanoparticles in aqueous media, without the need for any thermal treatment [18]. This approach was first demonstrated using lipase B from *Candida antarctica* (CAL-B) for the synthesis of Pd nanobiohybrids [25] and was subsequently extended to a broad range of metals and applications. Enzyme–copper nanobiohybrids were synthesized and applied in selective oxidation reactions and environmental remediation, including the degradation of Bisphenol A in water [26,27]. Enzyme-induced iron nanostructures of diverse morphologies, including nanorods, nanosheets, and nanorings, were obtained by simple mixing of enzymes with iron salts at room temperature, without requiring any reducing agent [28]. More recently, enzyme–metal hybrids incorporating Cu, Pd, Ag, and Mn were evaluated as artificial metalloenzymes mimicking catalase activity [29]. In all these systems, the enzyme plays a dual role: it acts as a structural scaffold controlling nanoparticle nucleation, size, and morphology, while, if its biological activity is preserved during synthesis, the resulting hybrid material can function as a bifunctional catalyst, coupling enzymatic and inorganic catalytic activity. This multifunctionality not only enhances overall catalytic performance but also broadens the potential applications of these nanobiohybrids in environmental remediation and other emerging technological fields.

The present work extends this nanobiohybrid concept to titanium-based materials for the first time, employing β-glucosidase as the protein scaffold in combination with TiCl_4_ and H_2_O_2_ in an aqueous bicarbonate buffer to produce brookite TiO_2_ nanostructures under entirely mild conditions and without any calcination step, and evaluating their performance as photocatalysts for environmental remediation, in particular in the degradation of Rhodamine B, a synthetic industrial dye used in textiles, paints, and plastics that is highly toxic to humans and the environment [30].

## 2. Materials and Methods

### 2.1. Materials

Lipase B from *C. antarctica* (CAL-B), catalase and β-glucosidase derived from *Aspergillus niger* (Novozyme 188) were supplied by Novonesis (Copenhagen, Denmark). Rhodamine B (RhB) was purchased from Merck (Darmstadt, Germany). Hydrogen peroxide (H_2_O_2_, 33%) and tetrahydrofuran (THF) were obtained from Panreac (Barcelona, Spain). TiCl_4_ was purchased from INSERT (Waltham, MA, US).

### 2.2. Bionanohybrid Characterization Techniques

Transmission electron microscopy (TEM) images were obtained on a JEOL JEM 1400 (120 kV), Jeol Ltd. (Tokyo, Japan). Aqueous suspensions of NPs were placed on 200-mesh copper grids coated with formvar/carbon without any staining.

The hybrids were characterized by X-ray diffraction (XRD), scanning electron microscopy (SEM) and spectrophotometric analysis. The XRD patterns were obtained using a PANalytical X’ Pert Pro polycrystalline diffractometer with a D8 Advance analysis texture configuration (Bruker, Billerica, MA, US) with a Cu Kα radiation (λ = 1.5406 A, 45 kV, 40 mA). To analyze the diffractograms, X’Pert Data Viewer and X’ Pert Highscore Plus software were employed. SEM imaging was performed with a TM-1000 microscope (Hitachi, Tokyo, Japan), and spectrophotometric analyses with a V-730 spectrophotometer (JASCO, Tokyo, Japan). Colloidal properties of the samples were studied in a Zetasizer Nano ZS TM, from Malvern Instruments, and carried out at pH 7, where the mean particle size was obtained based on the intensity distribution. Additionally, ζ-potential values were determined. Inductively Coupled Plasma-Optical Emission Spectroscopy (ICP-OES) was performed on an OPTIMA 2100 DV instrument (PerkinElmer, Waltham, MA, USA). Ten milligrams of the solid powder was treated with 5 mL of HCl (37% *v*/*v*) for acid leaching. Then 5 mL of water was added and centrifuged, and the clear solution was analyzed for metal content.

### 2.3. Synthesis of Ti Bionanohybrid

**Figure 1:**
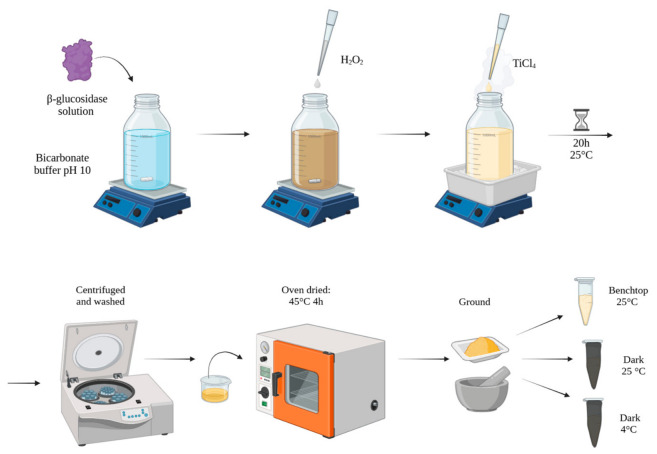
Schematic representation of the synthesis of β-glucosidase nanobiohybrids and **Ti@βGlu** and of the different storage conditions chosen to be evaluated.

A 2.1 mL measure of commercial β-glucosidase solution (19.59 mg/mL, determined by Bradford assay) was added to 60 mL of 1 M bicarbonate buffer (pH 10) (final enzyme concentration of 0.68 mg/mL) in a 100 mL glass bottle under magnetic stirring at room temperature under a fume hood (because of the Ti solution use). Then, 600 μL of H_2_O_2_ (33% *v*/*v*) was added to the enzyme solution slowly. After that, immediately, 350 μL of TiCl_4_ (1.71 mg/mL) was carefully added to the mixture, which was kept in an ice bath during the addition (Figure 1). *This precaution was necessary to avoid the violent exothermic reaction of TiCl_4_ with water. During this step, Cl_2_ or HCl gases were observed at the liquid surface; thus, the solution was left under the fume hood for several minutes*. It is important to store the remaining TiCl_4_ solution rapidly in an inert atmosphere to preserve reproducibility because it is extremely unstable. It could be observed that immediately after addition of the titanium precursor, the solution turned cloudy. Then, the solution was stirred at room temperature for 20 h. After that, the mixture was centrifuged at 8000 rpm for 15 min. The supernatant was discarded while the pellet was resuspended in distilled water. This washing procedure (centrifugation followed by resuspension) was repeated four times in total.

Finally, the pellet was resuspended in 5 mL of water and transferred to a crystallizer. The sample was oven-dried at 45 °C for 4 h. After that, a visible color change was observed: the material became light brown. The dried product was ground in a mortar to obtain a yield of 250 mg of a fine, homogeneous powder of the so-called **Ti@βGlu** catalyst. For stability studies, three different fractions of the final catalyst were created and stored under different conditions:

- In the dark, in a light-shielding Eppendorf;
- In the fridge at 4 °C, in a light-shielding Eppendorf;
- On the benchtop, in a regular 1.5 mL clear Eppendorf (Figure 1).

These samples were later compared to assess stability and relative performance over 15 days.

### 2.4. Photocatalytic Degradation of Rhodamine B

Photocatalytic activity of the hybrid was evaluated using Rhodamine B (RhB) degradation as a model reaction. Rhodamine B was chosen to evaluate photocatalytic activity due to its optical properties. It exhibits a strong absorption peak at λmax = 552.5 nm and an intense pink color; in solution, the color is visible even at very low concentrations. Photocatalytic degradation results in a significant decrease in absorbance at the absorbance maximum, accompanied by complete decolorization of the solution, allowing the reaction to be easily monitored visually and spectrophotometrically. A RhB solution with a concentration of 0.01 mM was prepared for all experiments. Different concentrations had been tested previously, but the one reported here was selected for practical reasons: it allowed direct measurement in the spectrophotometer without requiring dilutions and still provided absorbance values within a suitable range (0.05–1.0). This facilitated monitoring of the degradation over time and avoiding repeated dilutions, which could significantly increase experimental error. In addition, after the analysis, the solution could then be returned to the reaction vial to proceed with the investigated reaction. This ensured that the reaction volume and the catalyst/dye ratio remained constant throughout the whole experiment. The RhB solution was prepared by dissolving RhB in distilled water to obtain a final concentration of 0.01 mM, and stored in the dark at 4 °C. The solution remained stable for several months, and the same stock solution was used for all photocatalysis assays. To verify the photoactivity of the synthesized catalyst, the following setup was employed. Five milligrams of the catalyst **Ti@βGlu** was suspended in 2 mL of 0.01 mM RhB solution, and 20 μL of H_2_O_2_ was added to obtain a final concentration of 100 mM. Three vials were prepared, subject to different irradiation conditions:

1. Dark conditions: the vial was placed on a roller to ensure constant stirring and wrapped with multiple layers of aluminum foil to prevent light exposure.
2. Natural light: the vial was positioned in natural light, while being stirred on a roller.
3. UV irradiation: the vial was placed on a magnetic stirrer inside a dark chamber under an LED UV lamp (λ = 395 nm), 50 W, with a light intensity of 300–350 mW/cm^2^.

After three hours of reaction, the samples were centrifuged to separate the catalyst and obtain clear solutions. One milliliter of each supernatant was transferred into a plastic cuvette (2.5 mL capacity) and analyzed using a JASCO V-730 UV–Vis spectrophotometer (JASCO Corporation (Japan)). Spectra were recorded in the 390–600 nm range, and the maximum absorbance at 552.5 nm (corresponding to RhB absorbance maximum) was noted.

### 2.5. Evaluation of Optimal [H_2_O_2_] Concentration for the RhB Degradation Reaction

For this analysis, the experimental setup was as follows: 5 mg of **Ti@βGlu** was added to 2 mL of 0.01 mM RhB solution, to which varying amounts of H_2_O_2_ were added. In total, six different solutions were prepared, each containing a different concentration of hydrogen peroxide in order to evaluate the relative behavior. The prepared vials were placed on a magnetic stirrer inside a dark chamber, under a UV lamp (λ = 395 nm, LED 50 W). Absorbance measurements were performed after 3 h. This time point was selected because, in this interval, the differences in conversion between the various solutions (of different H_2_O_2_ concentrations) were sufficiently pronounced, allowing for a clear and reliable comparison of their relative efficiencies and enabling identification of the optimal concentration. After three hours, the solutions were centrifuged, and 1 mL of each sample was transferred into a plastic cuvette (2.5 mL capacity) and measured with the spectrophotometer in the 390–600 nm range. For each of the six samples, spectra were acquired and the maximum absorbance value at 552.5 nm (λ max of RhB) was recorded.

### 2.6. Preactivation Method by UV Lamp

For further investigation of the effect of UV exposure, the **Ti@βGlu** catalyst was first “incubated” under UV light at 395 nm (LED lamp 50 W) for 1 h in distilled water (5 mg catalyst in 2 mL water, stirred in a dark chamber). After this incubation, the suspension was centrifuged, the solid was recovered and the procedure described above was followed.

### 2.7. Recyclability

To evaluate the recyclability of the catalyst and its relative efficiency after each washing step, the chosen procedure for the RhB model was similar to the one described above. Five milligrams of catalyst and 2 mL of RhB solution (0.01 mM) were added to a vial containing H_2_O_2_ (100 mM), and the mixture was irradiated under UV light (λ = 395 nm, LED 50 W) for 1 h. After the reaction, the samples were centrifuged to separate the solid catalyst from the solution, whose UV-Vis spectra were then recorded in the 390–600 nm range. The maximum absorbance at λ = 552.5 nm was then measured.

Subsequently, the catalysts were washed with 1.5 mL of water and centrifuged for 2 min; this washing step was repeated again. After removal of the supernatant, the catalyst was reused in a new reaction cycle under the same conditions. For each cycle, 2 mL of fresh RhB solution (0.01 mM) and 20 μL of H_2_O_2_ (final concentration 100 mM) were added to a vial together with the recovered catalyst, and the mixture was irradiated under the same UV light for 1 h. The absorbance at λ = 552.5 nm was measured after each cycle. A total of five recycling cycles were carried out.

### 2.8. Radical Scavenging

To investigate the degradation mechanism of RhB and verify whether it follows a radical pathway, L-histidine was employed as a radical scavenger. L-histidine is known to be a quencher for both singlet O_2 and •OH [31]. Two solutions were prepared, each containing 2 mL of 0.01 mM RhB and 5 mg of catalyst Ti@βGlu and H_2_O_2_ (final concentration: 100 mM). These were left to react under UV light for 1 h, after which they were centrifuged and washed twice (according to the procedure described in Section 2.6), and catalysts were then recovered. The catalysts were added again, respectively, to 2 mL RhB and H_2_O_2_ 100 mM. In only one of the two solutions, 2 mg of L-histidine was added for a final concentration of 5 mM. The two solutions prepared (one with L-histidine, one without) were reused in a second RhB degradation test in UV light for 1 h under magnetic stirring. The solutions were centrifuged, and absorbance of supernatant was measured at λ = 552.5 nm. The results were analyzed to compare the behavior in the presence and absence of the scavenger.

### 2.9. Evaluation of One Month Benchtop Stability and in Different Storage Conditions

The stability of the catalyst over time was also investigated. To assess this, the synthesized catalyst, **Ti@βGlu**, was stored for 1 month on the benchtop in a plastic Eppendorf tube. After this period, it was tested again under the same experimental conditions used for the Rhodamine B model reaction, and the results were compared with those obtained for the freshly synthesized catalyst. Specifically, 5 mg Ti@βGlu of catalyst was suspended in 2 mL of 0.01 mM Rhodamine B solution, and H_2_O_2_ (100 mM) was added. After 3 h under UV, the solution was centrifuged, and the absorbance of the supernatant was measured at 552.5 nm. This value was used to compare the performance of stored versus freshly prepared catalysts. In addition, the stability of the catalyst under different storage conditions was evaluated (2.2.1) through the same RhB model just described. Specifically, the performance of the catalyst stored on the benchtop at room temperature (which was inevitably exposed to daylight) was compared with that of the same product stored for the same period in the fridge (4 °C), in a light-shielding Eppendorf at room temperature.

## 3. Results and Discussion

### 3.1. Synthesis and Characterization of Ti Bionanohybrids

First, different enzymes were used in the synthetic protocol as scaffolds: CALB lipase (monomer, 33 kDa), catalase (tetramer, 240 kDa) and β-glucosidase (monomer, 120 kDa), using TiCl_4_ as the Ti source in distilled water and NaOH to adjust the final pH to a value around 10. However, the strategy was not satisfactory, and water was replaced by a bicarbonate buffer to maintain good pH control throughout the whole synthesis. Better outcomes were observed here. In the earlier attempts, addition of TiCl_4_ caused a significant pH drop, leading to poor yields and unstable products. A 17% (*v*/*v*) measure of THF was then introduced in the synthetic route to better solubilize the titanium species. Additionally, the syntheses were both performed in the absence or in the presence of hydrogen peroxide. Eventually, it was only possible to obtain a stable catalyst, with a good yield, by employing the protein β-glucosidase. However, this method with THF in the synthesis revealed, after several tests of degradation of Rhodamine B, relatively low conversion values and adsorption of the compound to the material. Therefore, considering the above, we performed the synthesis in the same way but without the addition of THF to the solution, directly and exclusively in aqueous media. This route brought about the best results so far. From a 60 mL synthesis, approximately 250 mg of catalyst called **Ti@βGlu** was obtained. After grinding with a mortar, the material formed a fine white-yellow powder, which could be directly employed in the rhodamine degradation assay, where it exhibited good photocatalytic activity. Additionally, the solid product could be washed and centrifuged multiple times without significant loss of mass. Maintaining a constant pH throughout the process not only stabilized the reaction environment but also preserved the structural integrity of β-glucosidase essential for its role as a scaffold. Protein stability ensures that functional groups remain available for coordination of the growing titanium species, sustaining the templating effect necessary for nanobiohybrid formation. In the control experiments, where the synthesis was performed without the enzyme, almost no solid product (<5 mg) was obtained, demonstrating the necessary use of the protein as a scaffold to obtain a valid catalyst.

**Figure 2:**
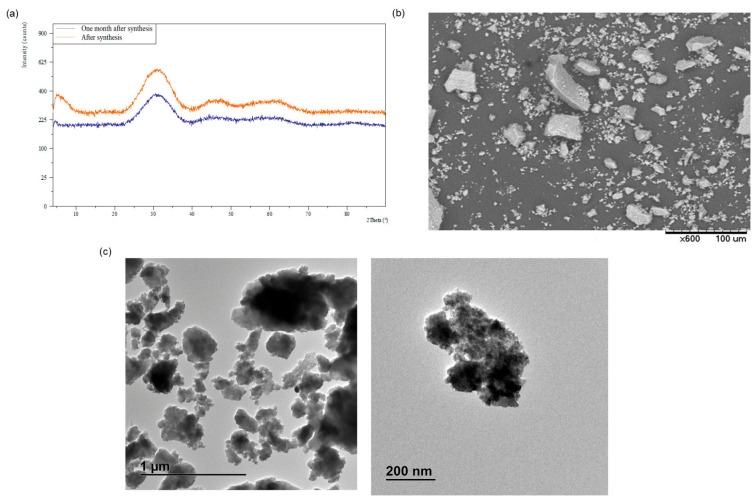
Structural Characterization of **Ti@βGlu**. (**a**) X-ray diffraction (XRD) pattern. (**b**) SEM image (scale bar: 100 µm). (**c**) TEM images (scale bars: left image: 1 µm, right image: 200 nm).

The X-ray diffraction (XRD) pattern presented in Figure 2a of **Ti@βGlu** reveals that the material exhibits low crystallinity, as indicated by the presence of broad diffraction bands and the absence of sharp, well-defined peaks associated with typical crystalline TiO_2_ phases. Furthermore, if any crystalline domains are present, the crystallite size is likely extremely small because of the very small intensity.

However, some peaks do show partial resemblance to brookite. The presence of brookite can usually be evidenced by the characteristic peak at 2θ = 30.8°, corresponding to the (121) plane [15]. Moreover, the other peaks at 2θ = 46° and 60° do match with the diffractogram of reference for brookite (JCPDS #00-029-1360) [15]. It is possible then to hypothesize the formation of the brookite crystalline form.

Additionally, a comparison between the patterns obtained immediately after synthesis and recorded one month later shows no significant structural changes (Figure 2a).

SEM analysis of the **Ti@βGlu** hybrid revealed a heterogeneous morphology characterized by irregular aggregates and variable particle sizes (Figure 2b). The material appears to form some clusters with larger agglomerates. SEM analysis was additionally performed to compare the morphology of the catalyst immediately after synthesis to that after one month of storage on the benchtop at room temperature. The sample after one month showed a similar appearance (data not shown).

Transmission electron microscopy (TEM) images showed the presence of nanoclouds, from around 100 nm to 700 nm (Figure 2c and Figure S1), demonstrating the nanostructured character of the material. Analysis of dynamic light scattering (DLS) corroborated this result, showing an average size of 482.2 nm with a stable colloidal dispersion and a zeta potential measurement of −46 mV (Figure S2), indicating a broad particle size distribution, so in turn a broad or multimodal population.

### 3.2. Photocatalytic Degradation of RhB

**Figure 3:**
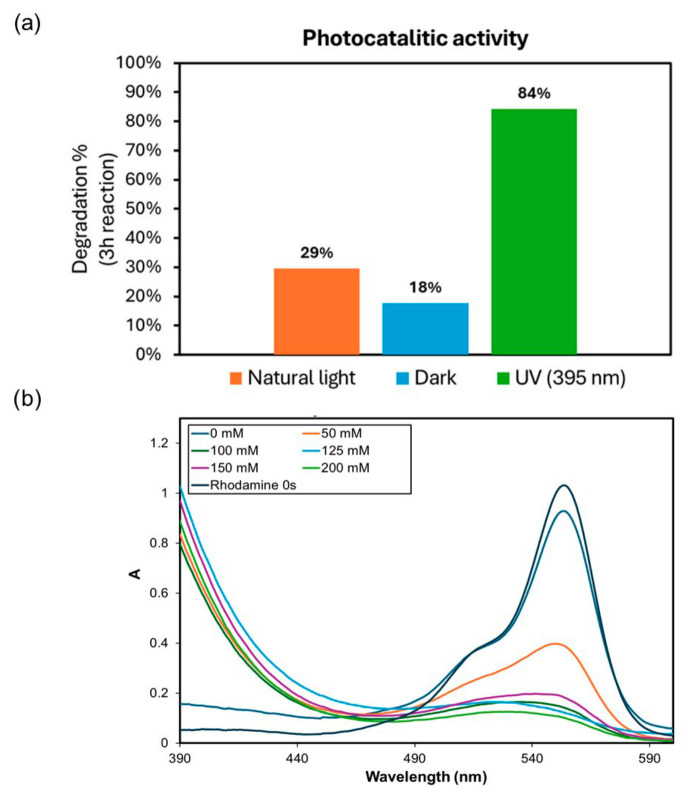
(**a**) Photocatalytic degradation of RhB (2 mL, 0.01 mM) under different lighting conditions after 3 h of reaction with 5 mg of catalyst and 100 mM H_2_O_2_. (**b**) Absorption spectra of RhB degradation (2 mL, 0.01 mM), after UV illumination, reacting with 5 mg catalyst and different hydrogen peroxide concentrations (0–200 mM). The dark blue line represents the blank of the absorbance of the RhB solution before the catalytic process (0 s). The curves were acquired after 3 h of reaction.

Initially, **Ti@βGlu** was evaluated in the degradation of RhB (0.01 mM) in water at room temperature under different illumination conditions (Figure 3). All experiments refer to a hydrogen peroxide concentration of 100 mM (Figure 3a). Reaction without a catalyst or directly using a free enzyme did not show any catalytic activity (data not shown).

As shown in Figure 3, after 3 h of reaction, the conversion catalyzed by **Ti@βGlu** under UV light was significantly higher, reaching a value of 84%, whereas the degradation process in natural light was 29%. In the dark, conversion was even slower than in natural light, with a value of 18%, and almost five times less than under UV light.

This clearly demonstrates that the activity enhancement is linked to exposure to a specific wavelength (395 nm), which corresponds to the theoretical band gap of the semiconductor. Therefore, photocatalytic behavior can be confirmed.

Additional tests were designed to investigate how lower H_2_O_2_ concentration could affect the reaction performance described above. H_2_O_2_ concentrations of 50 mM and 0 mM were tested. In these conditions, degradation at 3 h was reduced by 30% (60% instead of 84%) for the 50 mM solution. Instead, without hydrogen peroxide, after the same time interval, only slight conversion was observed (7%).

Then, to see the effect of higher concentrations of hydrogen peroxide, 125 mM, 150 mM and 200 mM H_2_O_2_ concentrations were tested. However, further increasing the concentration produced only minor improvements, with values approaching 85–90%. All measurements were taken at 552.5 nm (λ_max_ of RhB) after 3 h.

Figure 3 shows the UV–Vis absorption spectra (390–600 nm) recorded after 3 h of reaction for each concentration. Examination of the acquired spectra shows that higher degradation was accompanied by a progressive blue shift in the absorption maximum, indicating that changes in the dye were occurring. This suggests the effective degradation of RhB. Additionally, the appearance of a new absorption peak in the UV region suggests the formation of the dye’s degradation products, further supporting the successful activity of the catalyst.

### 3.3. Reutilization and UV-Preactivation Phenomenon

**Figure 4:**
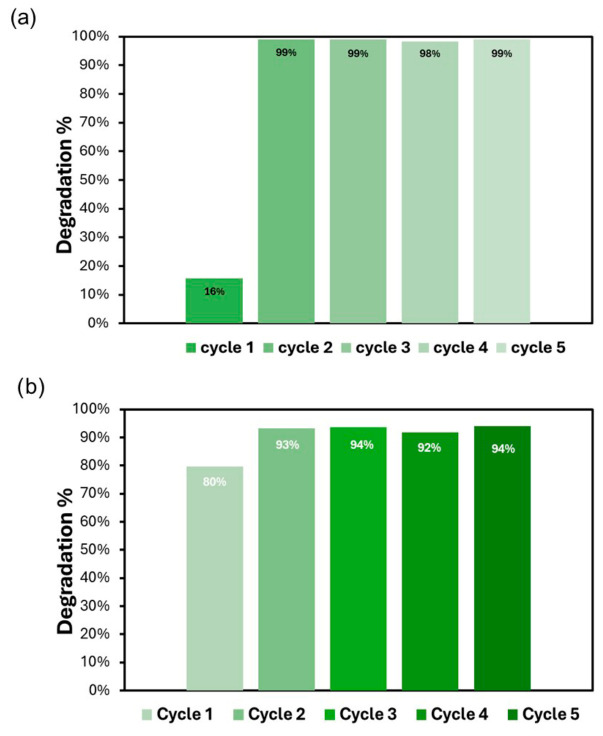
(**a**) RhB degradation percentage during five consecutive recycling cycles of **Ti@βGlu** under UV illumination. (**b**) RhB degradation during five consecutive recycling cycles of Ti@βGlu under UV-395 nm illumination. Here, the catalyst was pre-incubated for 1 h under UV-395 nm (LED 50 W) light in water before the standard reaction.

The recyclability of the **Ti@βGlu** catalyst was evaluated through repeated RhB degradation cycles under UV LED illumination. Each cycle lasted 1 h. Interestingly, in cycle two, a nearly complete degradation of the substrate was observed (Figure 4a) after only one hour, and the solution became completely clear. This enhanced performance persisted through the fifth cycle, indicating that the catalyst’s efficiency significantly increased after its initial UV exposure (Figure 4a). In order to demonstrate that the pretreatment with UV light before starting the reaction strongly affects the photodegradation capacity of the catalyst, the **Ti@βGlu** hybrid was first dissolved in an aqueous solution and placed under UV light.

Then, the catalyst was used in the RhB photodegradation. After this, the photodegradation conversion observed was 80% in a 1 h incubation, 5-fold higher than that observed without pretreatment (Figure 4b). This high conversion was even slightly increased after a second cycle of reusing the same catalyst, keeping the high efficiency intact after five cycles of reuse (Figure 4b), demonstrating the high recyclability capacity of this new Ti catalyst.

Additional tests were performed to verify whether the same conversion values could be obtained without washing in order to determine if any RhB degradation products were adsorbed on the catalyst surface, potentially reducing its activity by blocking the reactive sites. This was a way to better clarify the reason for increased activity after washing (data not shown).

The hypothesis that degradation byproducts accumulate on the catalyst surface and reduce its activity can be excluded for the following reasons. First, when the catalyst was reused without washing, its performance did not decrease significantly; on the contrary, high photoactivity was maintained, achieving up to 91% dye degradation. Second, if the observed behavior was solely due to adsorption and no UV-induced effects on the catalyst occurred, the experiment involving prior UV incubation in water would have resulted in low activity during the subsequent cycle, similar to the first cycle in the Rhodamine B reaction (approximately 16% degradation; see Figure 4a). Therefore, the hypothesis that surface hindrance by degradation products limits catalyst activity can be discarded.

### 3.4. Catalytic Mechanism of RhB Degradation

To investigate the degradation mechanism of RhB operated by the catalyst **Ti@βGlu** and define whether it proceeds via a radical pathway, L-histidine was used as a radical scavenger. L-histidine is known to quench singlet oxygen and hydroxyl radicals [31]. So if reactions were proceeding via formation of these two species, the degradation of RhB would not occur, or would occur to a minor extent, with the radicals being “subtracted” by histidine.

**Figure 5:**
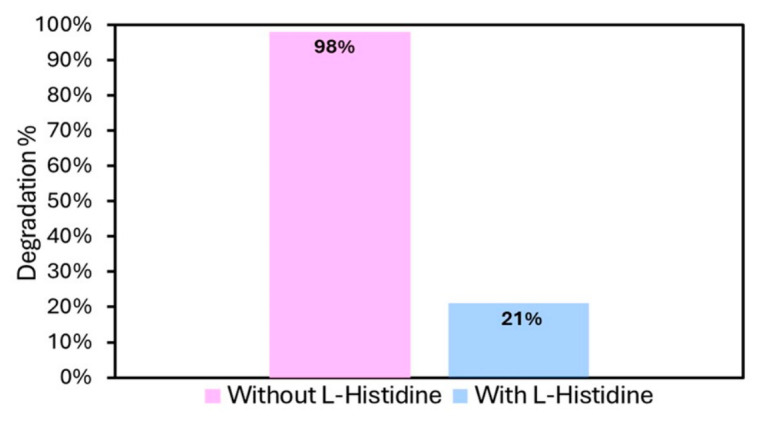
Catalytic RhB degradation in the presence or absence of L-histidine. The values shown correspond to absorbance of the solution after 1 h of “UV activation” and following 1 h of reaction (RhB 2 mL 0.01 mM, 100 mM H_2_O_2_, 5 mg catalyst, LED UV light 50 W, and 5 mM L-Histidine in one).

As shown in Figure 5, the effect of L-histidine in this experiment was clear: when absent, RhB degradation reached 98%, whereas in its presence, degradation dropped to 21%. This significant decrease confirmed that the photocatalytic process involves reactive oxygen species (ROS) and proceeds via a radical mechanism.

The photocatalytic degradation of RhB can then be defined to proceed via the generation of reactive oxygen species (ROS), initiated by the excitation of the semiconductor under UV light. Upon irradiation, electrons (e^−^) and holes (h^+^) are generated in the conduction and valence bands, respectively. These charge carriers participate in redox reactions that produce oxide radicals, which are responsible for the oxidative breakdown of RhB molecules. The general mechanism proposed in the literature suggests that electrons react with dissolved oxygen to form superoxide radicals and other ROS, while holes oxidize surface-bound water or hydroxide ions to generate additional reactive species. These ROS attack RhB molecules, leading to its breakdown and complete degradation [15].

Moreover, previous studies have shown that the addition of hydrogen peroxide (H_2_O_2_) accelerates the degradation process, which is consistent with its role as a promoter of •OH formation [23]. This observation aligns with our findings, where increasing concentrations of H_2_O_2_ led to higher RhB degradation, showing that H_2_O_2_ contributes to the generation of ROS and improves photocatalytic activity.

The specific mechanism of RhB photodegradation by the TiO_2_ hybrid in the presence of H_2_O_2_ is described further below:

TiO_2_  +  hv → e^−^ + h^+^ (1)

e^−^ + H_2_O_2_ → ·OH^−^ + •OH (2)

h^+^ + H_2_O_2_ → •O_2_H (3)

•OH + RhB → CO_2_  +  H_2_O  +  other products (4)

•O_2_H + RhB → CO_2_  +  H_2_O  +  other products (5)

Due to photoexcitation of the catalyst, electron–hole pairs were formed on the surface of TiO_2_ nanoparticles in the hybrid (Equation (1)). Then, in the presence of hydrogen peroxide, electrons in the conduction band are captured by it, breaking the molecule and generating hydroxyl radicals (Equation (2)). Additionally, holes in the valence band can also react with hydrogen peroxide: the hole extracts an electron from the hydrogen peroxide, oxidizing it. This generates hydroperoxyl radicals, which are highly reactive species (Equation (3)). Thus, the processes used to photodegrade RhB molecules are based on the possibility of the RhB molecules being directly degraded to small molecules by an attack of the active oxidative radicals (Equations (4) and (5)) [32,33].

In this process, the presence of L-histidine competed to trap the radicals, and therefore reduced the photodegradation of RhB in its presence, as we observed.

### 3.5. Stability

**Figure 6:**
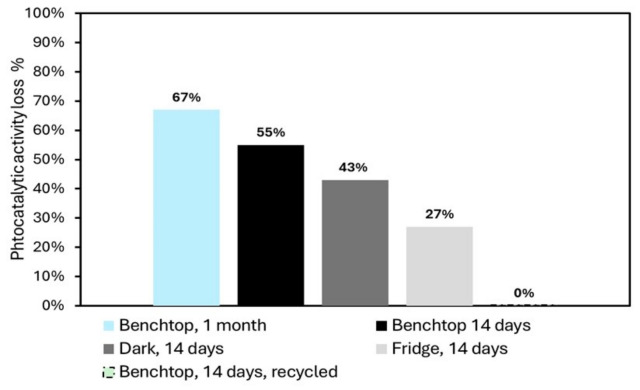
Photocatalytic activity loss (%) of **Ti@β-Glu** after storage under different conditions and incubation times. One month of incubation at 25 °C on the bench (blue), 14 days of storage on the benchtop at room temperature (black), 14 days in the dark at room temperature (dark gray), 14 days in the fridge at 4 °C (light gray), and recycled catalyst for 14 days on the benchtop at room temperature (green dashed line).

The long-term stability of the catalyst was evaluated by comparing its photocatalytic performance after storage under different conditions (Figure 6). The catalyst **Ti@β-Glu** was tested one month after synthesis, after being stored on the benchtop in a plastic Eppendorf tube at room temperature. The RhB degradation assay, as previously described, was used to assess activity, and the results were compared with those obtained using the freshly synthesized catalyst. As shown in Figure 6 (blue), storage under ambient conditions led to a 67% reduction in photocatalytic efficiency after one month. The stability test was then performed after storage of the catalyst under different conditions for 14 days: on the benchtop (exposed to light and at room temperature), in the dark at room temperature, and in the dark at 4 °C.

After two weeks of storage, the activity decreased under all conditions, with the most evident loss observed for the sample stored on the benchtop (55% loss of activity), followed by storage in the dark at room temperature (43% loss), while the sample refrigerated at 4 °C and in the dark catalyst preserved the highest activity (27% loss) (Figure 6, black, dark gray, and light gray, respectively).

These results indicate that exposure to light and ambient conditions accelerates deactivation of the catalyst, decreasing its photocatalytic activity. This result is coherent with what was obtained for one month of benchtop storage, describing a progressive decline in performance over time.

Additionally, to further investigate whether any reactivation was possible through recycling, the same catalyst stored on the benchtop was centrifuged, washed, and reused in a RhB degradation test following the recyclability protocol. Despite the previously observed decrease in activity after storage, the recycling procedure demonstrated a partial restoration of the catalyst’s initial performance. High degradation values were achieved (~90%, data not shown) within just 1 h, obtaining values even higher than the fresh catalyst. So the loss of activity due to benchtop storage could be recovered. This observation strongly suggests that UV irradiation induces modifications, improving photocatalytic efficiency, and restoring the negative effects produced by keeping the catalyst under natural light.

## 4. Conclusions

This work successfully demonstrated a sustainable and efficient strategy for synthesizing β-glucosidase/titanium nanobiohybrids to perform as efficient photocatalysts for environmental remediation. By employing β-glucosidase as a protein scaffold in combination with TiCl_4_ and H_2_O_2_ in a buffer with controlled pH, it was possible to obtain a functional hybrid material under mild conditions containing brookite TiO_2_ nanostructures without the need for calcination or energy-intensive steps and using a straightforward method.

The synthesized nanobiohybrids exhibited remarkable photocatalytic performance, achieving nearly complete degradation of RhB under UV light within one hour. The catalyst is capable of degrading RhB at a concentration approximately 36 times higher than the critical limit in water (0.14 ppm) [25]. The catalyst also showed excellent recyclability over five consecutive cycles and retained activity after storage. Radical scavenging experiments further revealed that the degradation mechanism involves reactive oxygen species (ROS), primarily hydroxyl and superoxide radicals, which is consistent with the expected photocatalytic pathway of Ti-based materials.

This performance opens up promising opportunities for practical applications, such as rapid wastewater treatment in textile and pharmaceutical industries, disinfection systems for healthcare environments, and portable purification devices. The combination of UV-LED activation and fast degradation kinetics defines these nanobiohybrids as an energy-efficient solution for environmental remediation and beyond.

Compared to conventional Ti-based nanoparticle synthesis methods, which often require high temperatures and long processing times, the bio-assisted approach presented here offers a simpler and more scalable alternative. The ability to control nanoparticle formation through enzymatic scaffolds allows for designing materials with on-demand properties.

The promising results obtained in this study highlight directions for future research. Exploring alternative titanium precursors could influence the structural and catalytic properties of the hybrids, potentially leading to improved or different performance. Achieving solar-driven activity would significantly broaden the potential of these nanobiohybrids as catalysts for water treatment and environmental remediation. Overall, this study provides evidence of the possibility of employing novel titanium bio-nanohybrid systems for photocatalytic treatment of waters and other environmental applications.

## Data Availability

The authors confirm that the data supporting the findings of this study are available within the article.

## Supplementary Materials

The following supporting information can be downloaded at: https://www.mdpi.com/article/10.3390/nano16130823/s1, Figure S1. TEM images of Ti@β-Glu. Figure S2. DLS analysis.

## References

[1] Yadav D., Karki S., Ingole P.G. (2022). Current advances and opportunities in the development of nanofiltration (NF) membranes in the area of wastewater treatment, water desalination, biotechnological and pharmaceutical applications. J. Environ. Chem. Eng..

[2] Amoatey P., Baawain M.S. (2019). Effects of pollution on freshwater aquatic organisms. Water Environ. Res..

[3] Sultana M.S., Islam M.S., Saha R., Al-Mansur M. (2009). Impact of the effluents of textile dyeing industries on the surface water quality inside D.N.D Embankment, Narayanganj. Bangladesh J. Sci. Ind. Res..

[4] Yusuf T.L., Orimolade B.O., Masekela D., Mamba B., Mabuba N. (2022). The application of photoelectrocatalysis in the degradation of rhodamine B in aqueous solutions: A review. RSC Adv..

[5] Wu C., Maurer C., Wang Y., Xue S., Davis D.L. (1999). Water pollution and human health in China. Environ. Health Perspect..

[6] Yaseen D.A., Scholz M. (2019). Textile dye wastewater characteristics and constituents of synthetic effluents: A critical review. Int. J. Environ. Sci. Technol..

[7] Ali H. (2010). Biodegradation of synthetic dyes—A review. Water Air Soil Pollut..

[8] Som I., Roy M., Saha R. (2020). Advances in nanomaterial-based water treatment approaches for photocatalytic degradation of water pollutants. ChemCatChem.

[9] Kumari H., Sonia, Suman, Ranga R., Chahal S., Devi S., Sharma S., Kumar S., Kumar P., Kumar S. (2023). A review on photocatalysis used for wastewater treatment: Dye degradation. Water Air Soil Pollut..

[10] Zhang J., Zhou P., Liu J., Yu J. (2014). New understanding of the difference of photocatalytic activity among anatase, rutile and brookite TiO_2_. Phys. Chem. Chem. Phys..

[11] Jeon J.P., Kweon D.H., Jang B.J., Ju M.J., Baek J.B. (2020). Enhancing the photocatalytic activity of TiO_2_ catalysts. Adv. Sustain. Syst..

[12] Gupta S.M., Tripathi M. (2011). A review of TiO_2_ nanoparticles. Chin. Sci. Bull..

[13] Zhang Y., Ge L., Ge S., Yan M., Yan J., Zang D., Lu J., Yu J., Song X. (2013). TiO_2_-graphene complex nanopaper for paper-based label-free photoelectrochemical immunoassay. Electrochim. Acta.

[14] Hanaor D.A.H., Sorrell C.C. (2011). Review of the anatase to rutile phase transformation. J. Mater. Sci..

[15] El-Sheikh S.M., Khedr T.M., Zhang G., Vogiazi V., Ismail A.A., O’Shea K., Dionysiou D.D. (2017). Tailored synthesis of anatase–brookite heterojunction photocatalysts for degradation of cylindrospermopsin under UV–Vis light. Chem. Eng. J..

[16] Nachit W., Ahsaine H.A., Ramzi Z., Touhtouh S., Goncharova I., Benkhouja K. (2022). Photocatalytic activity of anatase-brookite TiO_2_ nanoparticles synthesized by sol gel method at low temperature. Opt. Mater..

[17] Hirakawa T., Yawata K., Nosaka Y. (2007). Photocatalytic reactivity for O2•− and OH• radical formation in anatase and rutile TiO_2_ suspension as the effect of H_2_O_2_ addition. Appl. Catal. A Gen..

[18] Palomo J.M. (2019). Nanobiohybrids: A new concept for metal nanoparticles synthesis. Chem. Commun..

[19] Losada-Garcia N., Urriolabeitia E.P., Palomo J.M. (2023). Metal nanoparticles incorporated within graphene-enzyme preparations for synergistic multiactive catalysts. ACS Appl. Nano Mater..

[20] Ortega-Nieto C., Losada-Garcia N., Pessela B.C., Domingo-Calap P., Palomo J.M. (2023). Design and synthesis of copper nanobiomaterials with antimicrobial properties. ACS Bio Med Chem Au.

[21] Suvathi S., Ravichandran K., Karunakaran M., Praseetha P.K., Ayyanar M., Gobalakrishnan S. (2024). Biowastes-derived enzyme-powered zinc oxide and titanium oxide nanomaterials synthesis for anticancer and eco-friendly photocatalytic activity. Appl. Mater. Today.

[22] Suvathi S., Rathi R., Ravichandran K., Kavitha P., Ayyanar M., Praseetha P.K., Chidhambaram N. (2023). Improved photocatalytic dye degradation and seed germination through enzyme-coupled titanium oxide nanopowder—A cost-effective approach. Environ. Res..

[23] Wu Y., Tam T.V., Hur S.H., Rao P., Yoo I.K. (2023). Biomineralization of titanium dioxide with enhanced photocatalytic activity induced by lysozyme–polystyrene template. Mater. Chem. Phys..

[24] Nonoyama T., Kinoshita T., Higuchi M., Nagata K., Tanaka M., Sato K., Kato K. (2012). TiO_2_ synthesis inspired by biomineralization: Control of morphology, crystal phase, and light-use efficiency in a single process. J. Am. Chem. Soc..

[25] Filice M., Marciello M., Morales M.P., Palomo J.M. (2013). Synthesis of heterogeneous enzyme–metal nanoparticle biohybrids in aqueous media and their applications in C–C bond formation and tandem catalysis. Chem. Commun..

[26] Losada-Garcia N., Rodriguez-Otero A., Palomo J.M. (2020). Tailorable synthesis of heterogeneous enzyme–copper nanobiohybrids and their application in the selective oxidation of benzene to phenol. Catal. Sci. Technol..

[27] Losada-Garcia N., Rodriguez-Otero A., Palomo J.M. (2020). Fast degradation of Bisphenol A in water by nanostructured CuNPs@CALB biohybrid catalysts. Nanomaterials.

[28] Benavente R., Lopez-Tejedor D., Morales M.P., Perez-Rizquez C., Palomo J.M. (2020). The enzyme-induced formation of iron hybrid nanostructures with different morphologies. Nanoscale.

[29] Losada-Garcia N., Rodriguez-Otero A., Ortega-Nieto C., Azarmi A., Palomo J.M. (2022). Catalase like-activity of metal NPs–enzyme biohybrids. Appl. Nano.

[30] Skjolding L.M., Jørgensen L.v.G., Dyhr K.S., Köppl C.J., McKnight U.S., Bauer-Gottwein P., Mayer P., Bjerg P.L., Baun A. (2021). Assessing the aquatic toxicity and environmental safety of tracer compounds Rhodamine B and Rhodamine WT. Water Res..

[31] Wade A.M., Tucker H.N. (1998). Antioxidant characteristics of L-histidine. J. Nutr. Biochem..

[32] Costa E.P., Roccamante M.A., Amorim C.C., Oller I., Sanchez Perez J.A., Malato S. (2020). New trend on open solar photoreactors to treat micropollutants by photo-Fenton at circumneutral pH: Increasing optical pathway. Chem. Eng. J..

[33] Nosaka Y., Nosaka A. (2016). Understanding hydroxyl radical (·OH) generation processes in photocatalysis. ACS Energy Lett..

