# Supplementary material for: Novel Photo-Driven Activated Enzyme–Titanium Nanobiohybrids for Photocatalytic Applications
Source: Nanomaterials (Basel). 2026 Jul 4;16(13):823. doi: 10.3390/nano16130823 (PMC13363157; doi:10.3390/nano16130823)
Supplement: Supplementary file 1 [file nanomaterials-16-00823-s001.zip › nanomaterials-4281787-supplementary.pdf]

Supplementary information

Novel Photo-driven activated Enzyme-Titanium Nanobiohybrids for Photocatalytic Applications

Francesca Palla[1], Carla Garcia-Sanz[1], Marzia Marciello[2] and Jose M. Palomo*[,1]

[1] Instituto de Catalisis y Petroleoquimica (ICP), CSIC, Marie Curie 2, 28049, Madrid, Spain. E-mail: josempalomo@icp.csic.es

[2] Nanobiotechnology for Life Sciences Laboratory, Department of Chemistry in Pharmaceutical Sciences, Faculty of Pharmacy, Universidad Complutense de Madrid (UCM), Plaza Ramón y Cajal s/n, E-28040, Madrid, Spain

[3] Microscopy and Dynamic Imaging Unit, Fundación Centro Nacional de Investigaciones Cardiovasculares Carlos III (CNIC), Calle Melchor Fernández Almagro 3, E-28029, Madrid, Spain

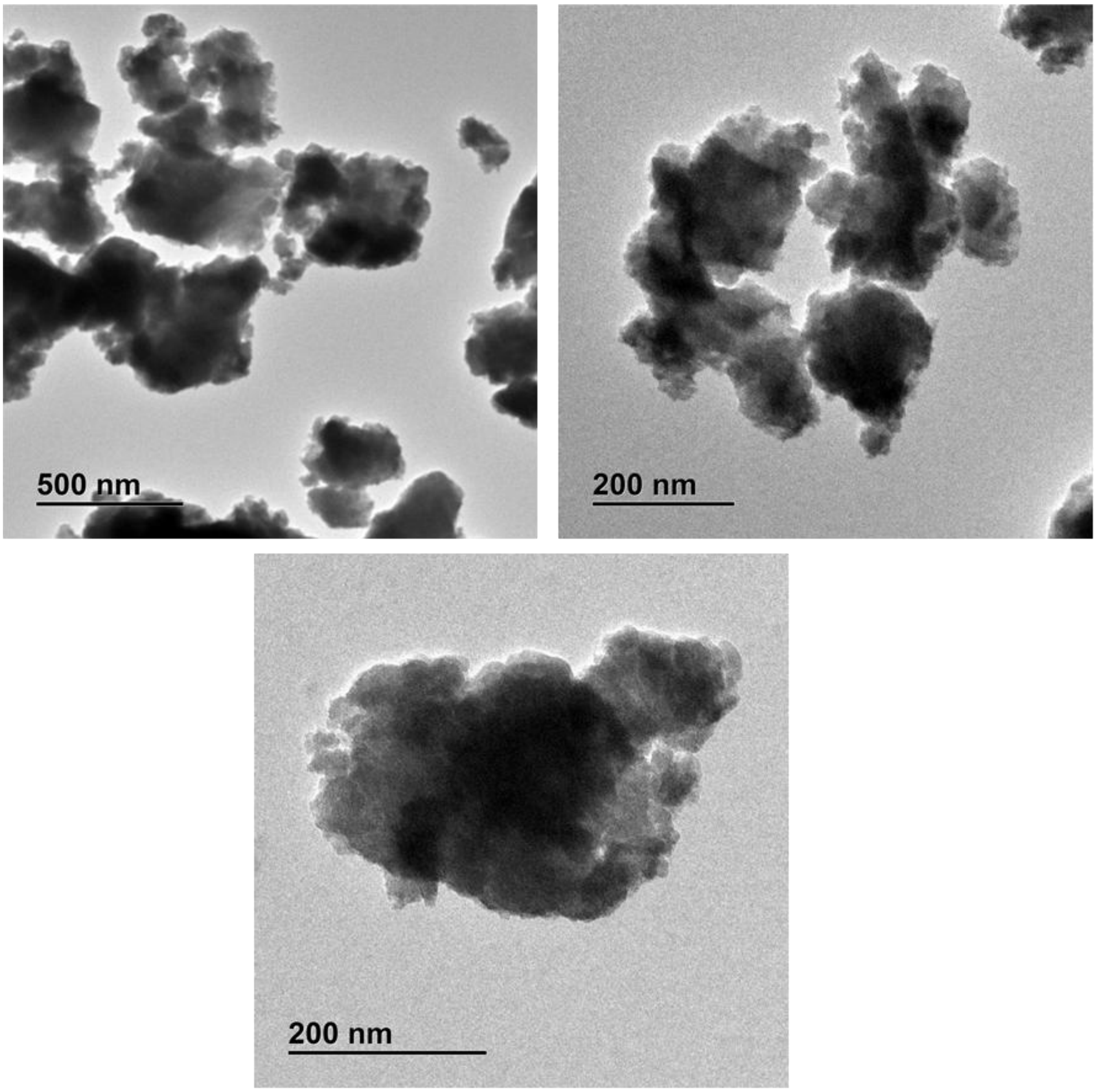

Figure S1. TEM images of **Ti@βGlu** hybrid.

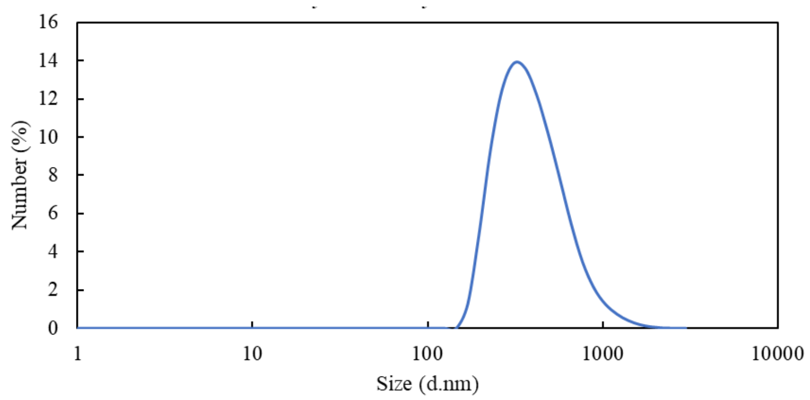

Figure S2. Particle size distribution obtained via DLS of **Ti@βGlu**.
